# JAK inhibitors improve ATP production and mitochondrial function in rheumatoid arthritis: a pilot study

**DOI:** 10.1007/s00296-023-05501-4

**Published:** 2023-11-20

**Authors:** Valentina Mihaylova, Maria Kazakova, Zguro Batalov, Rositsa Karalilova, Anastas Batalov, Victoria Sarafian

**Affiliations:** 1 Department of Medical Biology, Medical University-Plovdiv, Plovdiv, Bulgaria; 2 Research Institute at Medical University-Plovdiv, Plovdiv, Bulgaria; 3 Department of Propedeutics of Internal Diseases, Medical University-Plovdiv, Plovdiv, Bulgaria; 4 Clinic of Rheumatology, University Hospital “Kaspela”, Plovdiv, Plovdiv Bulgaria

**Keywords:** Rheumatoid arthritis, Mitochondrial function, Oxidative stress

## Abstract

Rheumatoid arthritis (RA) is a chronic, systemic autoimmune disease associated by inflammation of the synovial tissue and autoantibody production. Oxidative stress and free radicals are known to be indirectly implicated in joint damage and cartilage destruction in RA. Several studies describe the presence of mitochondrial dysfunction in RA, but few of them follow the dynamics in energy parameters after therapy. The aim of our investigation is to evaluate the direct effect of JAK inhibitors on cellular metabolism (and under induced oxidative stress) in RA patients. Ten newly diagnosed RA patients were included in the study. Peripheral blood mononuclear cells (PBMCs) and plasma were isolated before and 6 months after therapy with JAK inhibitors. A real-time metabolic analysis was performed to assess mitochondrial function and cell metabolism in PBMCs. Sonographic examination, DAS28 and conventional clinical laboratory parameters were determined also prior and post therapy. A significant decrease in proton leak after therapy with JAK inhibitors was found. The increased production of ATP indicates improvement of cellular bioenergetics status. These findings could be related to the catalytic action of JAK inhibitors on oxidative phosphorylation which corresponds to the amelioration of clinical and ultra-sonographic parameters after treatment. Our study is the first to establish the dynamics of mitochondrial parameters in PBMCs from RA patients before and after in vivo therapy with JAK inhibitors.

**Supplementary Information:**

The online version contains supplementary material available at 10.1007/s00296-023-05501-4.

## Introduction

Rheumatoid arthritis (RA) is a systemic autoimmune disease associated with inflammation of the synovial tissue, neovascularization and autoantibody secretion [1]. Genetic, environmental and immunological factors also contribute to the disease development and course. Recently, new data revealed the involvement of gut microbial imbalance (dysbiosis) in the pathogenesis of the disease. It has been linked to inflammation and autoimmune reactions in the joints [2]. The most important pathogenic features of RA progression are joint erosion and reduction of joint space [3]. In healthy individuals, the synovial membrane contains two types of synoviocytes—macrophage-like and fibroblast-like synoviocytes (FLS) with different characteristics [4]. These cells generally provide structural and dynamic joint integrity by controlling the composition of synovial fluid and the extracellular matrix. In RA, FLS show pathogenic properties by producing cytokines and proteases that support inflammation and cartilage destruction [5, 6]. T cells, B cells and macrophages are recruited in the synovial membrane. In the first stages of the disease, the immune tolerance of T cells to self-antigens is lost and the activation of antibody-producing B cells is enhanced. Subsequently, the aberrantly differentiated T cells acquire tissue-invasive functions, enter the joints and give rise to clinically significant arthritis. In the last stage, there is a tissue-destructive process that alters the joints and bones and a pannus is formed [7]. Pannus, like a tumour formation, increases the ATP deficit in the synovium vasculature and leads to poor tissue oxygenation and hypoxia [8]. In addition, the hypoxic microenvironment can initiate rapid cell proliferation and migration. The activation of immune cells requires a switch from oxidative metabolism, which is the normal state, to active glycolysis to maintain energy homeostasis. Some authors find a correlation between the level of oxygen in synovia and the severity of synovitis [9]. It is reported that hypoxia can cause multiple changes in mitochondrial structure, genome and kinetics, resulting in reduced ATP synthesis, excessive ROS production and accumulation of mtDNA mutations [10]. Studies show that damaged DNA triggers an innate immune response [11].

Mitochondrial dysfunction is observed in various tumour and autoimmune diseases, including RA. It is characterised by several features, such as reduced efficiency of oxidative phosphorylation, decreased production of ATP, loss of maintenance of the electrical and chemical transmembrane potential of the inner mitochondrial membrane and alterations in the function of the electron transport chain (ETC) [12]. Changes in mitochondrial respiration might mediate pathogenic mechanisms in inflammatory diseases. It is known that mitochondrial dysfunction represents the switch of cellular metabolism from oxidative phosphorylation to glycolysis. High speed of ATP production promotes accelerated cell proliferation [13]. Furthermore, endogenous stressors (pro-inflammatory cytokines, oxidative stress, etc.) or environmental factors can alter mitochondrial function and increase the risk of RA [14]. Inflammatory mediators produced by activated chondrocytes, osteocytes and infiltrating immune cells trigger intracellular processes that can change mitochondrial activity [15]. The complex interplay between inflammation and mitochondrial dysfunction might be implicated in RA pathogenesis. In recent years, numerous studies have demonstrated that inflammation, associated with altered immune system function, may explain the heterogeneous clinical picture in RA [16–18].

Improvements in diagnosis of RA have been achieved but precise, definitive therapy and reliable monitoring are still missing. The continuous search for new treatment strategies widened the therapeutic arsenal with the application of biological anti-rheumatic targeted synthetic small molecules—JAK inhibitors. In-depth pharmacokinetic studies indicated the application of dosage forms with the highest possible binding affinity to the target protein [19]. Still, the use of approved drugs often leads to side effects, such as hypertension, liver failure, slow response time to biological therapy and a high risk of infections. In this context, methods for direct delivery of active substances using nanotechnology are discussed. The bioavailability of drugs via several mechanisms includes enhancing the solubility and permeability reduces the treatment dose [20].

There are biomarkers reflecting the clinical condition of patients after therapy with conventional pharmaceuticals, but there are few studies on the effect of targeted treatment on mitochondrial function [21]. A very limited number of investigations report imbalance in the energy metabolism of cells and the presence of mitochondrial dysfunction in RA patients in parallel with therapy [22].

The goal of our study is to evaluate cellular metabolic profile and key parameters of mitochondrial function in RA patients before and after therapy with JAK inhibitors and to search for an association between cell energy metabolism and disease progression.

The current investigation uses the most innovative and advanced technology to determine the mitochondrial status of living cells in real time. It is aimed at examining multiple key mitochondrial parameters to assess the effect of in vivo treatment of patients with RA on cell metabolism. It presents novel data showing dynamics in proton leak and production of ATP after therapy with JAK inhibitors indicating recovery of cellular bioenergetics status. A relationship with improvement of clinical and ultra-sonographic parameters after treatment is observed.

## Methods

### Patients

Ten newly diagnosed RA patients according to the ACR/EULAR 2010 [23] at the Rheumatology Clinic of University Hospital “Kaspela” were enrolled in the study. The mean age of the participants (females—8 and males—2) was 55 ± 14years. All participants signed informed consent. The study was carried out in compliance with the Declaration of Helsinki.

Venous blood samples were obtained before and six months after therapy with JAK inhibitors. Conventional biochemical and immunological markers, such as rheumatoid factor (RF) and anti-CCP antibodies, as well as acute-phase reactants—erythrocyte sedimentation rate (ESR) and C-reactive protein (CRP), were examined before and after therapy. The JAK inhibitors (Tofacitinib and Upadacitinib) were administered in their optimal approved daily dose—5 mg twice a day, according to the general characteristics of the therapeutic and the recommendations of the manufacturer.

Several composite scores are available to assess the activity of RA. We applied the most commonly used score system—the Disease Activity Score 28 (DAS28), which assesses 28 joints to evaluate patients’ clinical status.

Patients were examined by DAS28 prior to and after therapy. DAS28 is based on counts of tender and swollen joints, the patient’s global assessment of disease activity, and ESR or CRP. The criteria for separating remission, low, moderate and high disease activity are scores of 2.6; 3.2; and 5.1 respectively [24].

### Isolation of peripheral blood mononuclear cells (PBMCs)

EDTA venous blood (6 ml) was obtained from each patient, in compliance with all conditions for venipuncture. The centrifugation was for 10 min at 1800 rpm. The buffy coat layer was mixed with 2 ml of phosphate-buffered saline (PBS) (pH = 7.4) and layered onto Histopaque (Sigma-Aldrich, *d* = 1.077 g/ml) at a ratio of 1:1, using density gradient centrifugation. It was then centrifuged for 30 min at 1800 rpm. The layer with PBMCs was aspirated and washed twice with 10 ml PBS, centrifuged at 1800 rpm for 10 min. PBMCs were cultured in RPMI-1640 medium (Gibco CAT#P04-18000) supplemented with 10% FBS and 1% penicillin/streptomycin. They were grown overnight on 24-well plates in an incubator at 37 °C, 5% CO_2_ and high humidity. Cell viability and number were determined using a “LUNA” automated cytometer (Logos Biosystems, Anyang, Korea). For metabolic assays, cells were brought to a final concentration of 2 × 10^5^ cells per well with RPMI-1640 in 8-well Seahorse microplates. Assessment of cell viability and number was performed immediately before metabolic analysis.

### Ultrasound examination

Ultrasound (US) assessment of wrists, hands and forefoot was conducted using a GE Logic E9 machine with a ML6-15-D Matrix Array linear probe for the joint/tendon assessment. Two-dimensional US (B-Mode/Gray scale US) and Power Doppler US were performed. *GSUS* frequency was 11–15 MHz depending on the examined joint and GSUS gain was estimated based on joint regions and patients with an average value of 50%. The following settings were used for *PDUS*: frequency 8.3 MHz; pulse repetition frequency 600–800 Hz; PDUS gain in relation to joint regions and patients with an average value of about 50%; low wall filter. The German US7 score of Backhaus et al. [25] was applied.

We examined by GSUS and PDUS 7 joints of the clinically dominant hand/foot, affected more by swelling or tenderness, using the German US7 score (wrist, second and third MCP and PIP, second and fifth metatarsophalangeal (MTP) joints. Several parameters were evaluated according to the definitions and standardised protocols of Outcome Measures in Rheumatology (OMERACT), including the presence of synovitis, tenosynovitis and erosions [26].

All investigated structures were evaluated by GSUS and PDUS. Tenosynovitis/paratenonitis and erosions were documented as present (1) or absent (0). The scoring range was 0–27 for GSUS synovitis, 0–7 for GSUS tenosynovitis/paratenonitis, 0–39 for PDUS synovitis, 0–21 for PDUS tenosynovitis/paratenonitis and 0–14 for erosions scores. US7 score was calculated as the sum of the synovitis score, tenosynovitis/paratenonitis score, and erosion score on GSUS and of the synovitis and tenosynovitis scores on PDUS.

### Assessment of mitochondrial function by Mito Stress test

The Mito Stress test detects oxygen consumption (OCR), an indicator of mitochondrial respiration of living cells in real time. The bioenergetic profile is determined by five parameters, such as ATP production, proton leak, maximum respiratory capacity (MRC), spare respiratory capacity (SRC), and non-mitochondrial oxygen consumption (NMOC). The plates were hydrated with double distilled water and PBMCs were cultured on the first day after venipuncture. On the next day, after assessment of cell viability and number of the cultured cells, they were seeded on hydrated plates at a concentration of 2 × 10^5^ cells/ml in Seahorse XFp base medium pH 7.4 (Agilent CAT#103576-100). PBMCs were visualised using an inverted microscope before analysis to check steady distribution in the wells. The PBMCs were analysed in triplicates in a series of consecutive runs with the strictest execution of the same experimental procedure. All PBMCs were isolated and processed the same way. They were counted immediately after isolation, which was then immediately followed by bioenergetic quantitation. Each bioenergetic experiment was performed in triplicates. Data from the patients were statistically processed in triplicate prior to and after therapy. Representative curves illustrating mitochondrial respiration before and after therapy for each patient are provided in the Supplementary Fig. 1.

Mitochondrial respiration was examined in real time after application of inhibitors. Basal OCR measurements were performed before ATP synthase was inhibited using oligomycin to measure the mitochondrial ATP production. After treatment of the cells with FCCP, maximum mitochondrial respiration and spare respiratory capacity were determined. Finally, addition of rotenone and antimycin A allows the evaluation of non-mitochondrial respiration rates (Supplementary Fig. 2). The role and the effect of the inhibitors on mitochondrial activity are presented in Supplementary Table 1.

Another plate with cells was incubated with 2,3-dimethoxy-1,4-naphthalenedione (DMNQ) for 1 h. DMNQ brings in free oxygen radicals in the cell and provides conditions to determine mitochondrial function under oxidative stress. The assessment of mitochondrial activity was conducted by a metabolic test based on the measurement of oxygen consumption by living cells in real time via Seahorse analyzer (Agilent).

### Statistical analysis

Data from the patients were statistically processed—each performed in triplicate prior to and after therapy. Differences between the obtained data before and after therapy were evaluated for significance using *t* test for non-parametric data and Wilcoxon test for two related samples. Spearman’s correlation coefficient was calculated to assess the link between the studied variables. The level of statistical significance of the null hypothesis was *p* < 0.05. Statistical analysis was carried out with the SPSS software version 19.0.0. Data obtained from Seahorse (Bioscience, Agilent) were analysed using the Wave software.

## Results

### Assessment of clinical and laboratory parameters

To evaluate the role of mitochondrial activity in RA, the patients were examined for disease activity in parallel with clinical and laboratory parameters. Possible associations between the main variables of interest were analysed before and after therapy. The results are presented in Table 1.

**Table 1:** Clinical and laboratory parameters in RA patients before and after therapy with JAK inhibitors

Parameters	Before therapy Mean (min.–max.)	After therapy Mean (min.–max.)
CRP mg/L, (< 5)	31.04 (5.1–124.0)	5.72 (1.0–16.8)
RF IU/ml, (< 10)	252.5 (27.4–885.0)	59.09 (25.90–137.0)
ESR mm/h, (< 15)	57.80 (25.0–100.0)	29.40 (15.0–45.0)
Anti-CCP-Ab IU/ml, (< 17)	948 (33.12–4782)	23.87 (17.30–30.44)
DAS28, remission (≤ 2.6) Low disease activity (> 2.6–3.2) Moderate disease activity (> 3.1–5.1) High disease activity (> 5.1)	5.847 (3.86–7.41)	3.415 (2.51–5.11)
GUS7 score, (0–108)	25.7 (9.0–46.0)	11.50 (7.0–16.0)

Data are presented as the median ± standard deviation by SPSS

*CRP* C-reactive protein, *RF* rheumatoid factor, *ESR* erythrocyte sedimentation rate, *Anti-CCP-Ab* anti-cyclic citrullinated peptide-antibody, *DAS28* diseases activity score, *GUS7* Ultrasound score

A significant correlation with the parameters of interest was revealed. A positive correlation between DAS28 and GUS7 score (**p < 0.0001, *z* = 0.918) was found, as well as between DAS28 and CRP (**p = 0.005, *z* = 0.802), before therapy. An association between ESR and CRP (*p = 0.042, *z* = 0.683) was detected, after therapy.

### Mitochondrial activity in PBMCs incubated with DMNQ before and after therapy with JAK inhibitors

**Fig. 1:**
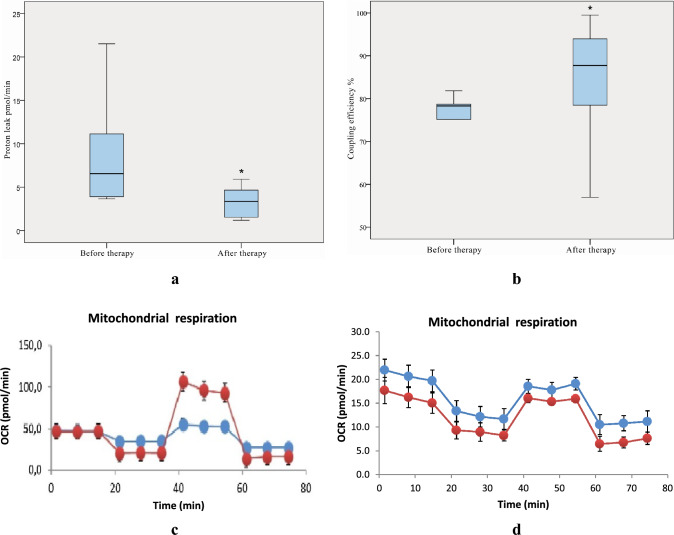
Proton leak (**a**) and coupling efficiency % (**b**) in PBMCs, incubated with DMNQ, before and after therapy with JAK inhibitors. Representative curves of mitochondrial respiration in one RA patient, before (**c**) and after (**d**) therapy with JAK inhibitors

A significant decrease in proton leak after therapy with JAK inhibitors was detected compared to the level of proton leak before therapy (*p = 0.021) (Fig. 1a).

In addition, we measured the amount of ATP production normalised to basal respiration, presented as coupling efficiency %. A difference after treatment was detected as values of the bioenergetic parameter increased significantly (*p = 0.037). The dynamics in ATP levels is illustrated in Fig. 1b.

Representative curves from one RA patient are presented in Fig. 1c, d. Representative curves for all patients are included in Supplementary Fig. 1.

### Oxygen consumption in conditions of lacking and induced oxidative stress

Three indicators reflecting oxygen consumption and capacity were examined: MRC, SRC and NMOC. There was a difference between all these parameters before and after therapy but statistically no significant. To test our concept, the same experiment was performed in parallel on PBMCs, but they were incubated with DMNQ. The results showed a statistically significant decrease in cell’s ability to meet the energy needs. Increase in non-mitochondrial respiratory consumption in conditions of induced oxidative stress was detected. The results are presented in Table 2.

**Table 2:**
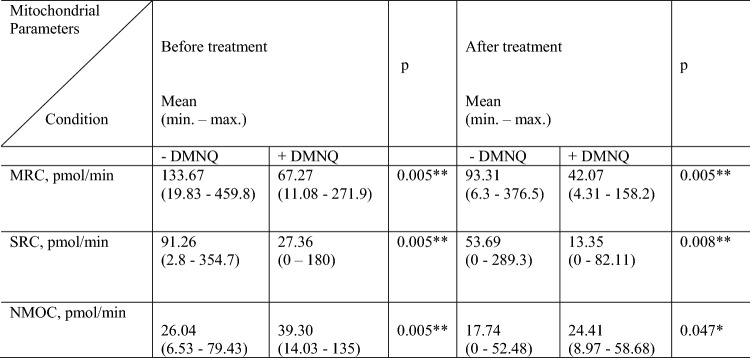
Mitochondrial parameters determined in condition of absent or induced oxidative stress in PBMCs in RA patients, before and after therapy with JAK inhibitors

*MRC* maximal respiratory capacity, *SRC* spare respiratory capacity, *NMOC* non-mitochondrial oxygen consumption, *DMNQ* 2,3-Dimethoxy-1,4-naphthoquinone

Wilcoxon signed-ranks test *p < 0.05; **p < 0.01 by SPSS

### Correlations between clinical, laboratory and mitochondrial parameters after therapy

Additionally, a positive correlation between non-mitochondrial respiration and CRP (*p = 0.043, *z* = 0.700) was found. An association between non-mitochondrial respiration and RF (*p = 0.035, *z* = 0.636) and a relationship between proton leak in oxidative conditions and RF (*p = 0.032, *z* = 0.645) was also identified.

Data are expressed as mean ± SD from curves of mitochondrial respiration of all patients and presented in box plots (Fig. 1a, b). Coupling efficiency % is a parameter that is software calculated in percentages based on ATP and basal respiration values from the curves obtain by Seahorse analyzer.

Representative curves from one RA patient are presented in Fig. 1c, d. The curves show oxygen consumption in PBMCs of RA patient in real time in both conditions (in absent and present DMNQ—2,3-Dimethoxy-1,4-naphthoquinone).

## Discussion

The pathogenesis of RA is associated with severe oxidative stress, which causes numerous post-translational modifications of proteins and leads to changes in the bioenergetic profile of mitochondria [27]. In addition, the altered state of the cells provokes mitochondria to work at a higher energy level to maintain the proliferative effect. This leads to metabolic and immune dysfunction related to autoimmunity [28].

Our study is the first to establish the dynamics of mitochondrial parameters in PBMCs from RA patients before and after in vivo therapy with JAK inhibitors upon induction of oxidative stress, via DMNQ.

In a previous survey, we examined ten age-matched healthy controls. The levels of basal respiration, maximal and SRC in RA patients before therapy were significantly higher compared to the control group (Supplementary Figure—3). These changes are likely to be related to disease activity and elevated energy needs [29].

The current investigation reveals a reduction in proton leak after therapy with JAK inhibitors compared to naïve patients. Probably, our results reflect the restoration of the inner mitochondrial membrane and reduction of oxidative stress. We could assume that this is due to the beneficial effect of the targeted drug on the membrane potential [30].

We did not find any published scientific data on JAK inhibitors’ effect on mitochondrial function, in the context of RA, although proton leak is associated with several pathological conditions. Lopes de Melo et al. reported higher levels of proton leak in PBMCs of patients with type I diabetes than in healthy controls. [31] High values of proton leak are detected in CD4 + cells in the acute phase of *T. cruzi*-infected mice, associated with excessive cellular production of ROS, possibly leading to cellular dysfunction and cell death [32]. We determined the coupling efficiency % which is composed of the ATP produced, normalised to the cells’ basal oxygen consumption, and expressed as a percentage. Significantly higher levels of ATP synthesis were measured after therapy compared to the values before therapy. These results can be related to the catalytic action of JAK inhibitors on oxidative phosphorylation which is associated with reduction of ROS and a switch from glycolysis to oxidative metabolism [16].

The correlation between clinical assessment scores of disease severity and laboratory parameters is essential for therapy monitoring and disease. Ultrasonography is suggested to be a sensitive method that provides information about soft tissue, articular effusion, bone surface and hyaline cartilage [33]. The advantages of ultrasonography assessment are the early detection and the excellent monitoring of subtle disease progression [34]. The German US7 score combined in one point system changes related to soft tissue and bone erosion, together with DAS28 which assesses 28 joints, is a routine method used for monitoring of disease activity [35, 36]. The ultrasound examination showed a significant reduction in joint inflammation during treatment with JAK inhibitors. A decrease in the values of both GSUS and PD modality, respectively the US7 score, was registered. We determined that increased ATP values corresponded with improvement in clinical and laboratory tests, such as ESR, anti-CCP, CRP and RF, as well as with DAS28 and GUS7 scores, after therapy. This suggests that the amount of ATP produced may serve as an indicator of disease monitoring. McGarry et al. found that primary synovial fibroblasts from RA patients cultured with JAK inhibitors induced increased ATP levels and decreased ROS [37]. In accordance with our study, the authors performed metabolic investigation by Seahorse analyzer. We did not find any significant differences in maximal and reserve respiratory capacity before and after therapy. However, its dynamics was established after the administration of the redox cycling agent. Following the introduction of additional oxygen radicals, mitochondrial parameters dropped by more than 50%. Their decrease is believed to correlate with depletion of cell viability and cell function [38].

A similar effect on reserve respiratory capacity was reported in cardiac myocytes, cultured in hypoxic conditions [39]. We assume that JAK inhibitors might have a protective function on PBMCs and did not induce additional oxidative stress, thus allowing the cells to meet the increased energy needs. Metabolic adaptation is dependent on mitochondrial function and uses the mitochondrial reserve to satisfy higher energy demands. Amongst mitochondrial respiratory parameters, SRC represents a particularly robust functional parameter for assessing mitochondrial reserve [40]. Probably the adaptation of mitochondria during the treatment period with JAK inhibitors, and especially under oxidative stress exhausts SRS. This effect might be due to depletion of substrates in the cell since the reserve capacity requires both glucose and fatty acids [39]. It was revealed the oxidative phosphorylation, and the MRC significantly increased after in vitro treatment of synovial explants and fibroblasts from RA patients with JAK inhibitors [37]. In addition, macrophages in RA patients, because of the upregulation of glycolysis and oxidative phosphorylation, consume more oxygen, and more ATP is produced [16]. However, research on mitochondrial dysfunction in PBMCs after therapy is very limited. In our study, a decrease in the MRC after therapy in some patients was detected. We hypothesised that the findings reflected individual dynamics in energy metabolism and systemic patient immune response.

Non-mitochondrial respiration is an expression of the amount of oxygen that continues to be consumed by cellular enzymes, even after the blockage of the electron transport chain, and serves as an important indirect indicator for measuring mitochondrial respiration. The parameter is related to promoted glycolysis in case of strong oxidative stress. In our investigation, we show a drop in non-mitochondrial respiration as absolute value after therapy and significantly higher levels when oxidative stress is induced. After the addition of the DMNQ agent, we observed induction of mitochondrial dysfunction and a shift to the glycolytic pathway for ATP production. Balogh et al. demonstrated for the first time a metabolic shift towards glycolysis upon induction of oxidative stress in synovial fibroblasts [27]. No relationship between clinical laboratory biomarkers and mitochondrial activity has been reported in the literature yet. Our study comes across an interesting finding that demonstrates a feeble association between non-mitochondrial respiration in oxidative conditions and markers for inflammation as CRP and RF, which are used in routine clinical practice. Furthermore, we observed a relationship between proton leak and RF suggesting that the excessive production of ROS and mitochondrial dysfunction might trigger different inflammatory pathways. We could speculate that biochemical markers can indirectly assess mitochondrial function and disease activity in RA.

However, the present study has several limitations. Given the unique and expensive method of assessing mitochondrial activity and the still limited number of patients followed up before and after therapy, the data need further confirmation on a larger patient cohort. This would strengthen the preliminary results of our pilot study and will give a possible impetus to the management of RA. Another limitation is related to the single choice of therapy. In the future studies, a comparison with conventional therapeutic approaches (e.g. Methotrexate) is required.

## Conclusion

In conclusion, novel data detecting mitochondrial dysfunction in isolated PBMCs from RA patients are presented. Cell metabolic parameters and ultrasonographic results partially recover after therapy with JAK inhibitors highlighting the future perspectives for monitoring RA treatment based on parallel analysis of mitochondrial function, ultrasonography and clinical laboratory parameters.

### Supplementary Information

Below is the link to the electronic supplementary material.

Supplementary file1 (PDF 493 KB)

Supplementary file2 (PDF 28 KB)

Supplementary file3 (PDF 492 KB)

Supplementary file4 (PDF 74 KB)

## Data Availability

All data are available from the corresponding author on reasonable request.
