# Supplementary material for: JAK inhibitors improve ATP production and mitochondrial function in rheumatoid arthritis: a pilot study
Source: Rheumatol Int. 2023 Nov 20;44(1):57–65. doi: 10.1007/s00296-023-05501-4 (PMC10766792; doi:10.1007/s00296-023-05501-4)
Supplement: Supplementary file 1 — Supplementary file1 (PDF 493 KB) [file 296_2023_5501_MOESM1_ESM.pdf]

Supplementary Figure 1

Mitochondrial respiratory curves of PBMCs in RA patients before and after therapy with JAK-inhibitors

PBMCs in RA patients - before therapy

PBMCs in RA patients - after therapy with JAK-inhibitors

Patient 1

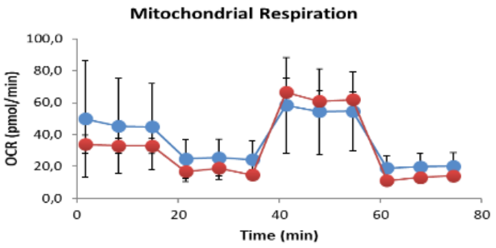

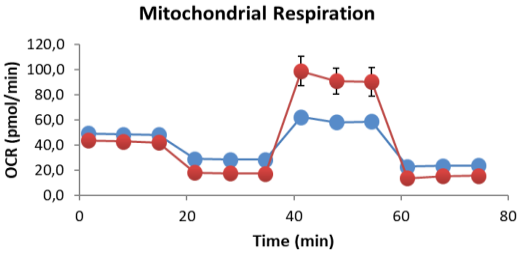

Patient 2

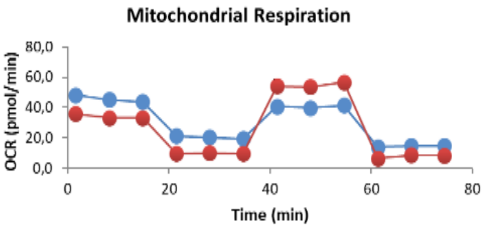

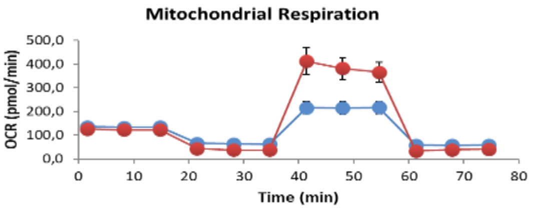

Patient 3

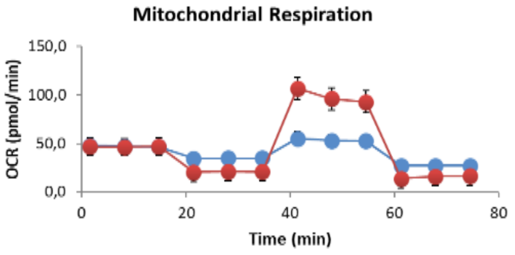

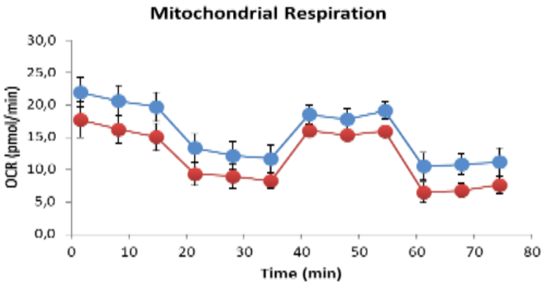

Patient 4

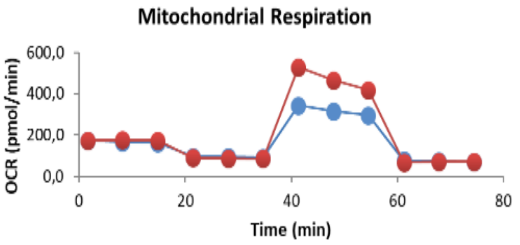

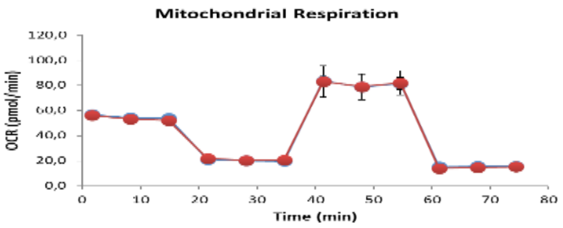

Patient 5

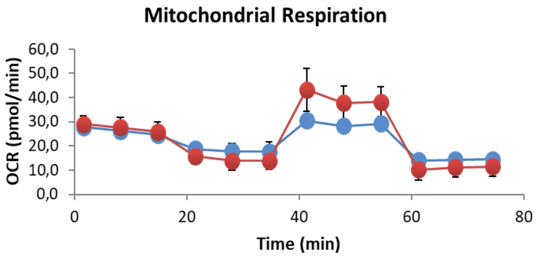

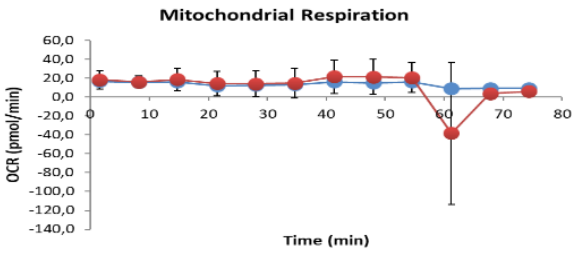

Supplementary Figure 1

Mitochondrial respiratory curves of PBMCs in RA patients before and after therapy with JAK-inhibitors

PBMCs in RA patients - before therapy

PBMCs in RA patients - after therapy with JAK-inhibitors

Patient 6

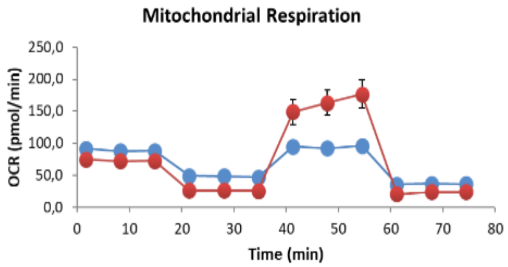

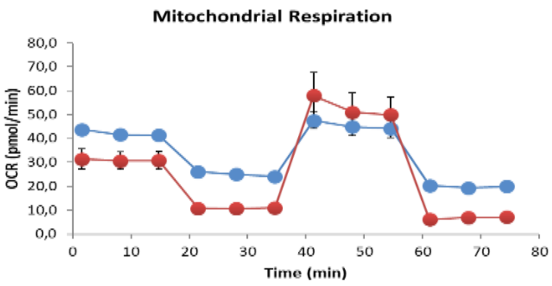

Patient 7

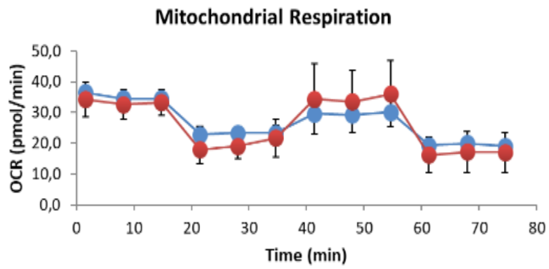

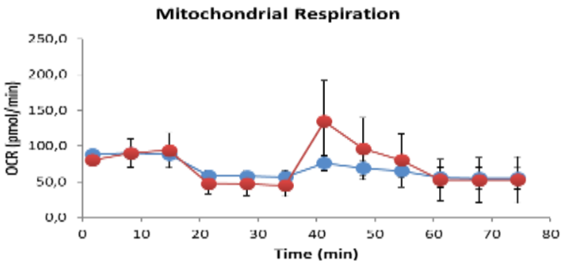

Patient 8

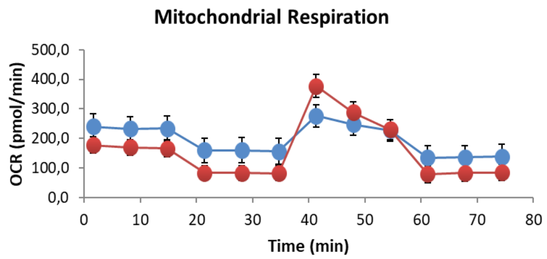

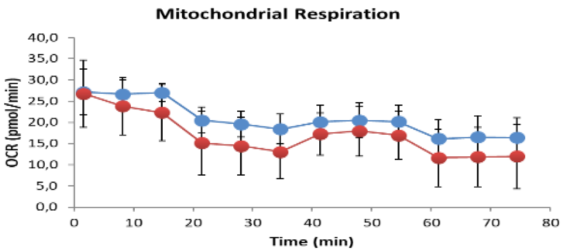

Patient 9

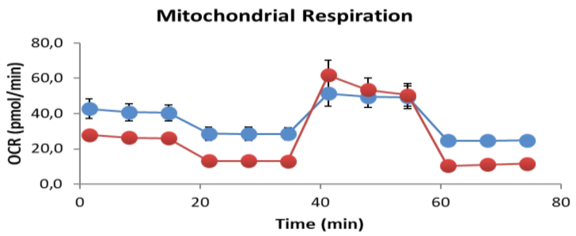

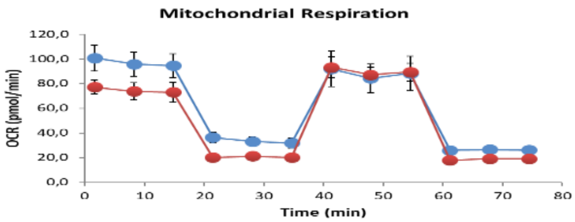

Patient 10

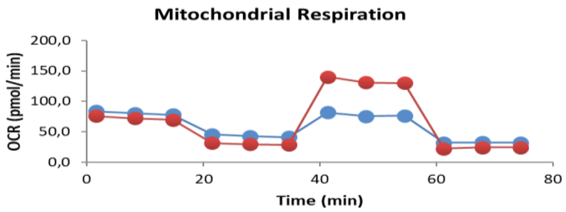

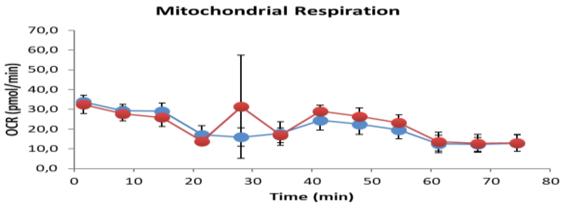

Red line – Oxygen consumption of PBMCs in RA patients

Blue line - Oxygen consumption of PBMCs in RA patients incubated with DMNQ
