# Supplementary figures and images for: JAK inhibitors improve ATP production and mitochondrial function in rheumatoid arthritis: a pilot study
Source: Rheumatol Int. 2023 Nov 20;44(1):57–65. doi: 10.1007/s00296-023-05501-4 (PMC10766792; doi:10.1007/s00296-023-05501-4)

## Seahorse XF Cell Mito Stress Test Profile

### Mitochondrial Respiration

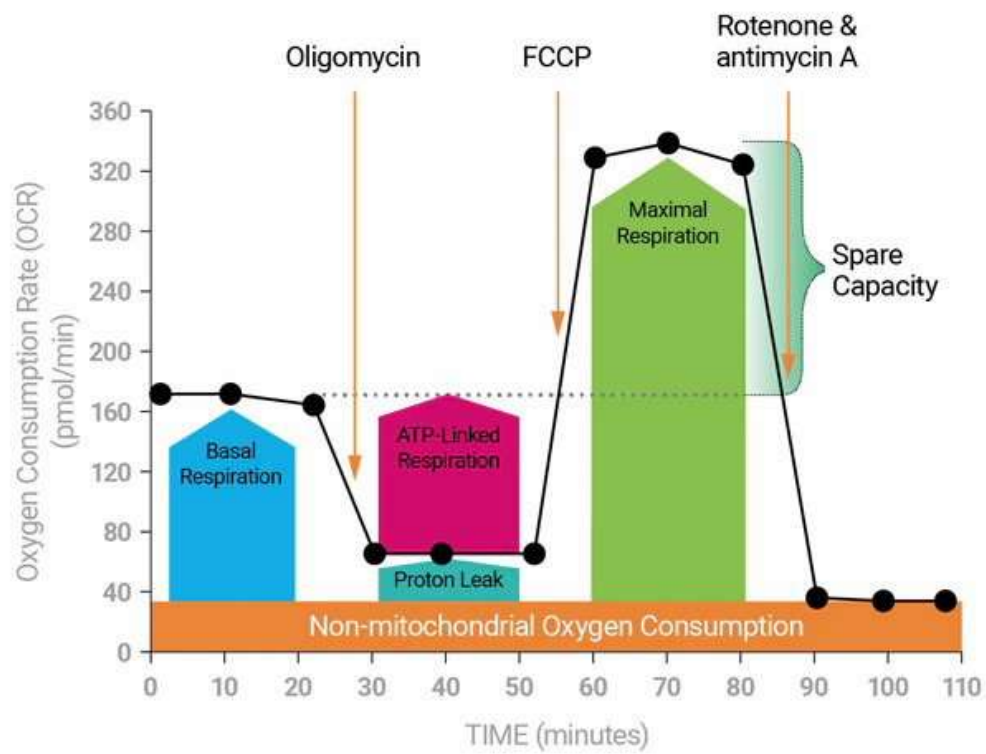

Supplement: Supplementary file 2 — Supplementary file2 (PDF 28 KB) [file 296_2023_5501_MOESM2_ESM.pdf]
