# Supplementary material for: JAK inhibitors improve ATP production and mitochondrial function in rheumatoid arthritis: a pilot study
Source: Rheumatol Int. 2023 Nov 20;44(1):57–65. doi: 10.1007/s00296-023-05501-4 (PMC10766792; doi:10.1007/s00296-023-05501-4)
Supplement: Supplementary file 3 — Supplementary file3 (PDF 492 KB) [file 296_2023_5501_MOESM3_ESM.pdf]

Supplementary Figure 2
Mitochondrial respiratory curves of PBMCs in healthy controls

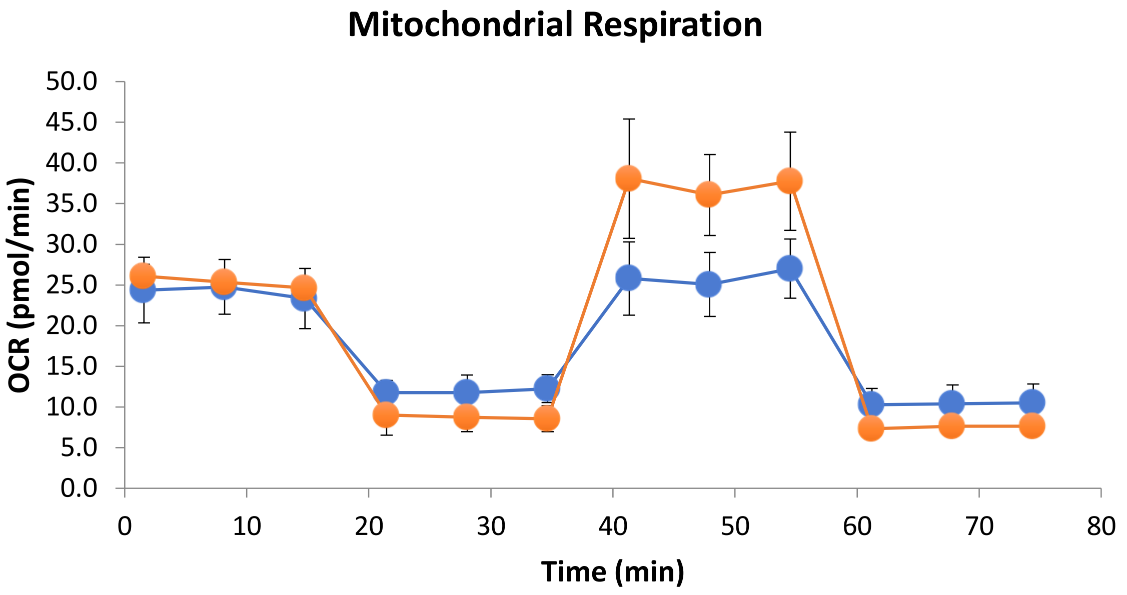

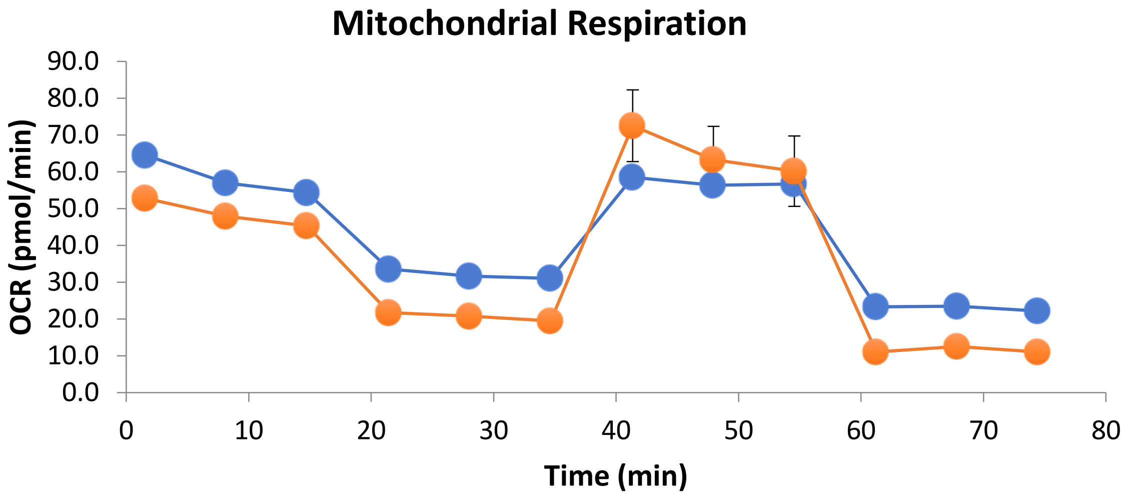

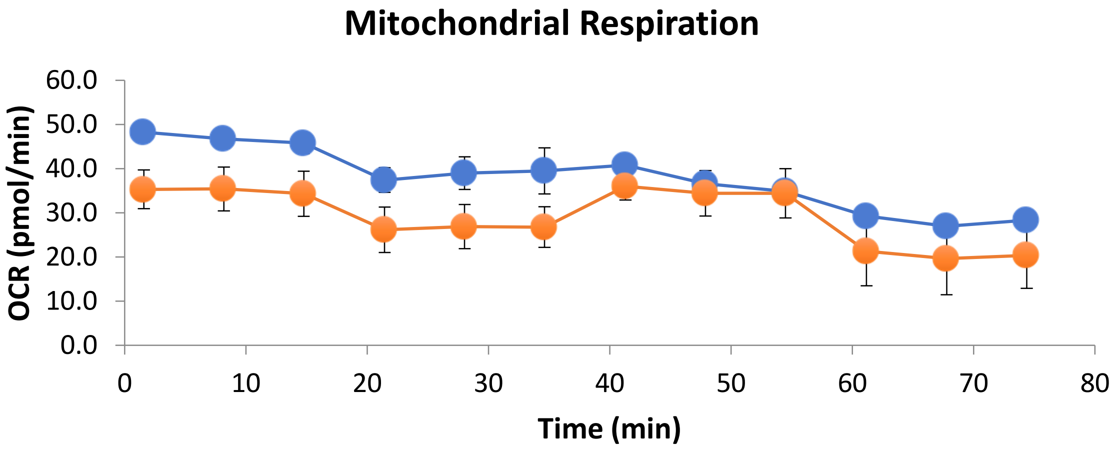

Supplementary Figure 2
Mitochondrial respiratory curves of PBMCs in healthy controls

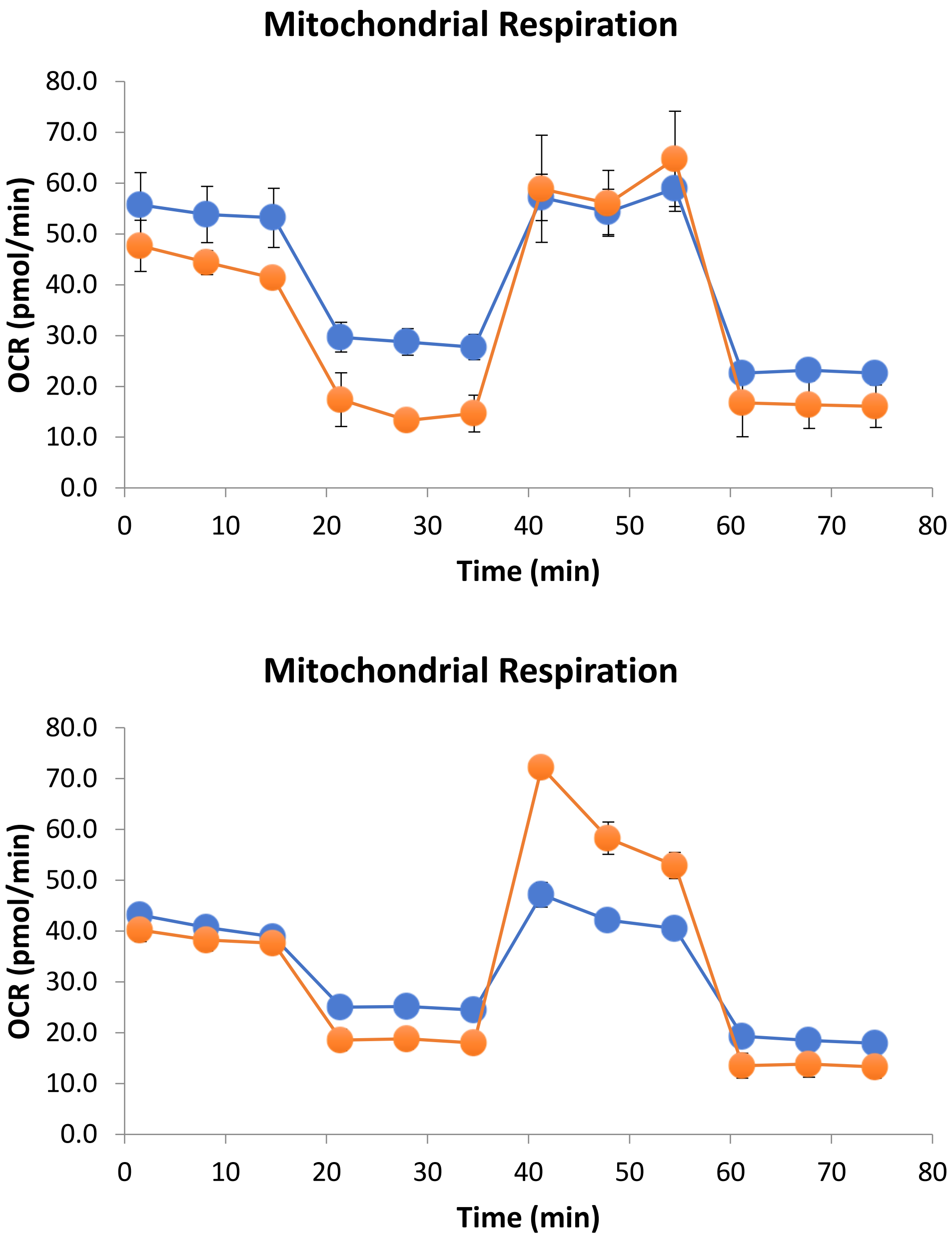

Orange line – Oxygen consumption of PBMCs in healthy controls
Blue line - Oxygen consumption of PBMCs in healthy controls incubated with DMNQ
