# Supplementary material for: JAK inhibitors improve ATP production and mitochondrial function in rheumatoid arthritis: a pilot study
Source: Rheumatol Int. 2023 Nov 20;44(1):57–65. doi: 10.1007/s00296-023-05501-4 (PMC10766792; doi:10.1007/s00296-023-05501-4)
Supplement: Supplementary file 4 — Supplementary file4 (PDF 74 KB) [file 296_2023_5501_MOESM4_ESM.pdf]

Supplementary Tab. 2 - Role and effect the inhibitors on mitochondrial activity

Inhibitor	Role	Effect
Oligomycin	Inhibits ATP synthase, reduces electron flow through the electron transport chain (ETC)	Decreases ATP levels
FCCP<br>Carbonyl cyanide 4-(trifluoromethoxy)phenylhydrazone	Disrupts mitochondrial membrane potential.	Increases the rate of cellular oxygen consumption
Rotenone	Inhibits complex III of the ETC	Suppresses mitochondrial respiration
